# Supplementary material for: The serogroup B meningococcal outer membrane vesicle-based vaccine 4CMenB induces cross-species protection against Neisseria gonorrhoeae
Source: PLoS Pathog. 2020 Dec 8;16(12):e1008602. doi: 10.1371/journal.ppat.1008602 (PMC7748408; doi:10.1371/journal.ppat.1008602)
Supplement: S1 Table — (DOCX) [file ppat.1008602.s001.docx]

**S1 Table. Amino acid identity between proteins of *N. meningitidis* MC58 and *N. gonorrhoeae* FA1090**

| Protein | ID^a^/MC58 | ID^b^/FA1090 | AA^‡^/MC58 | AA^‡^/FA1090 | % AA identity |
| --- | --- | --- | --- | --- | --- |
| PilQ | AAY52170 | Q5FAD2 | 769 | 723 | 94 |
| BamA | AAF40639 | Q5F5W8 | 797 | 792 | 95 |
| MtrE | AAF42061 | NG1363^2^ | 467 | 467 | 96 |
| PorB.1B | NP275030 | YP208842 | 331 | 348 | 70 |

^a^Accession ID from <https://www.ncbi.nlm.nih.gov/protein/>; ^b^Gene ID number from <http://stdgen.northwestern.edu/>; ^‡^AA, number of amino acids in the protein. MC58, *N. meningitidis* strain MC58; FA1090, *N. gonorrhoeae.*
