# Supplementary figures and images for: The serogroup B meningococcal outer membrane vesicle-based vaccine 4CMenB induces cross-species protection against Neisseria gonorrhoeae
Source: PLoS Pathog. 2020 Dec 8;16(12):e1008602. doi: 10.1371/journal.ppat.1008602 (PMC7748408; doi:10.1371/journal.ppat.1008602)

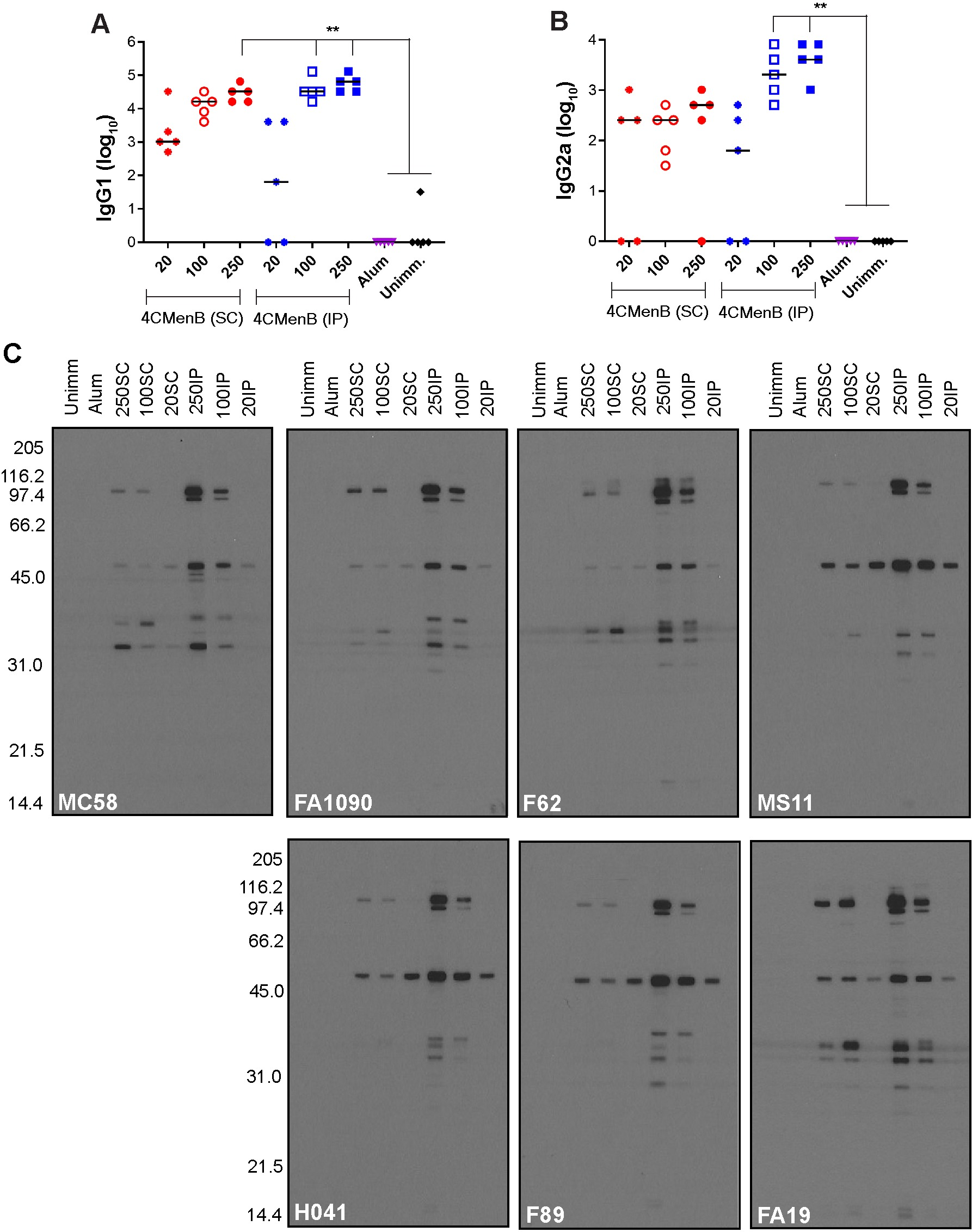

Supplement: S1 Fig — Groups of 5 BALB/c mice were given 20, 100 or 250 μl of the formulated vaccine on days 1 and 28 by the IP or SC routes. (A,B) Serum IgG1 and IgG2a titers using microtiter plates coated with the formulated 4CMenB vaccine 10 days after the second immunization. A dose response is shown for serum IgG1 in both IP- and SC-immunized mice. (C) Serum reactivity (1:5,000) against whole cell lysates of Nm strain MC58 and of 6 different Ng strains using anti-mouse IgG secondary antibody shows a similar dose response based on differences band intensity. A nonparametric test (Kruskal Wallis with Dunn’s multiple comparison) was used to analyze ELISA data due to the low sample size. **, p < 0.01. (TIF) [file ppat.1008602.s002.tif]

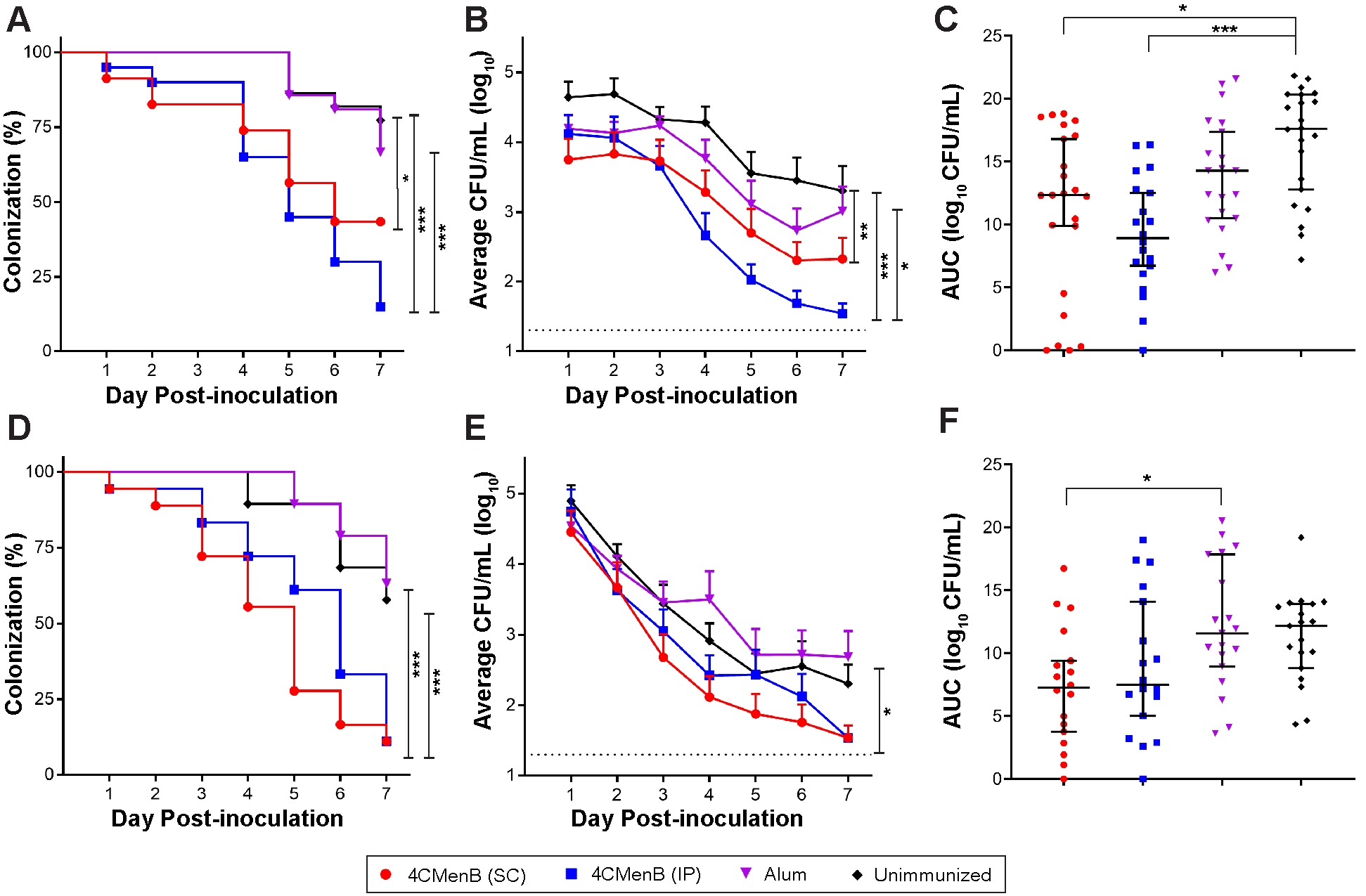

Supplement: S2 Fig — In each experiment, mice were immunized three weeks apart with 250-μl doses of 4CMenB by the IP (blue) or SC (red) route or given PBS (black) or alum (purple) by the IP route (n = 25 or 20 mice per group in experiments 1 (A-C) and 2 (D-F), respectively). Three weeks after the final immunization, mice were challenged with Ng strain F62 as described in the Methods. (A,D) Percentage of culture-positive mice over time and average CFU per ml of a single vaginal swab suspension, respectively for experiment 1 (n = 20–23 mice/group); (B,E) Percentage of culture-positive mice over time and average CFU per ml of a single vaginal swab suspension, respectively for experiment 2 (n = 18–19 mice/group); (C,F) Total bioburden recovered from individual mice over 7 days expressed as area under the curve (AUC) for experiments 1 and 2, respectively. The median with 95% CI is indicated for each group.*, p < 0.05, **p < 0.01, *** p < 0.0001. (TIF) [file ppat.1008602.s003.tif]

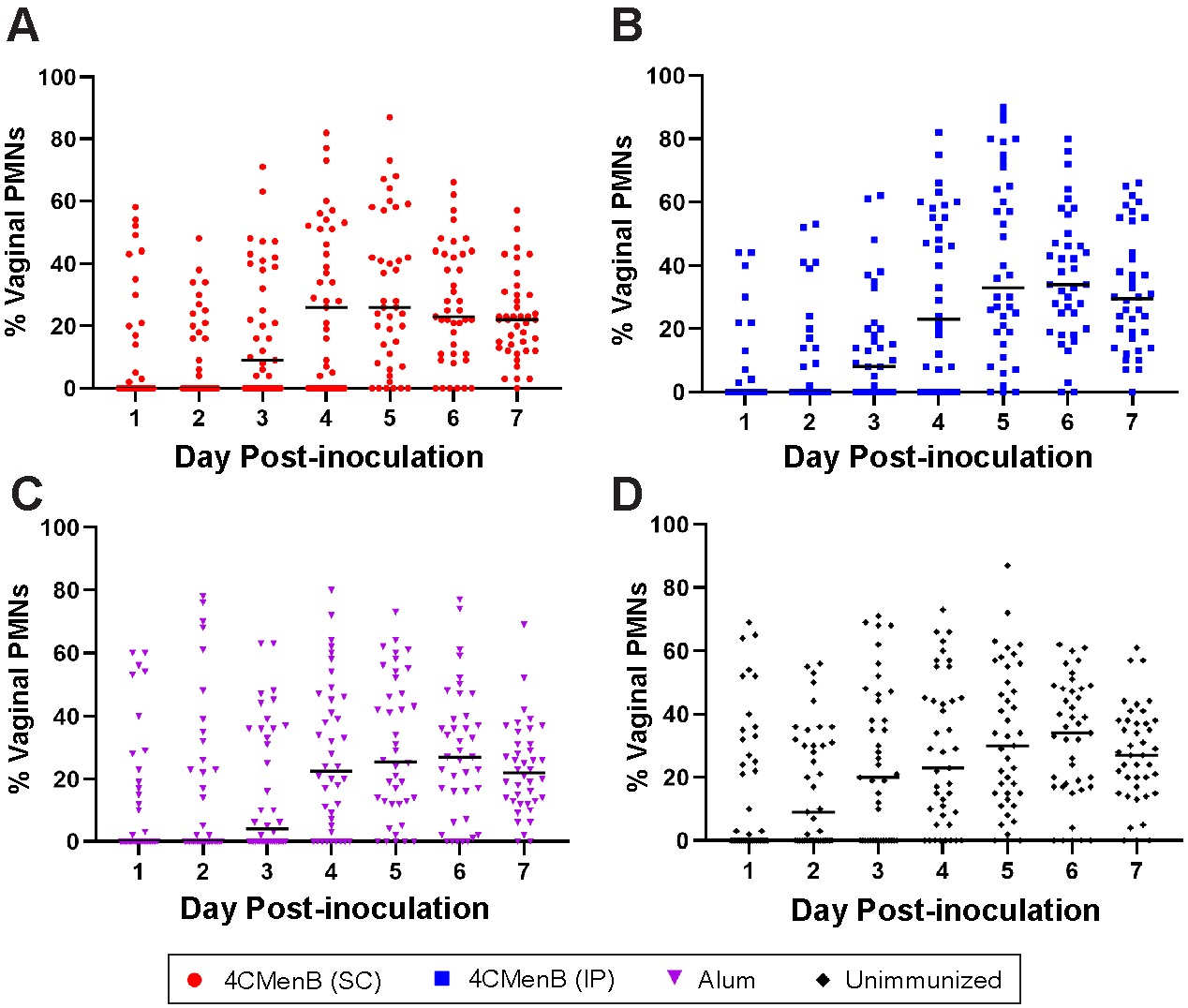

Supplement: S3 Fig — Vaginal smears collected on each culture day following bacterial challenge were stained with Hemacolor Stain (Sigma), and the percent of PMNs among 100 vaginal cells was determined by cytological differentiation using light microscopy. An increase in the percentage of PMNs occurred between days 4–7 as is characteristic of this model, with no statistical difference between the groups. The median percent PMNs is shown by the horizontal bar for each time-point. (TIF) [file ppat.1008602.s004.tif]

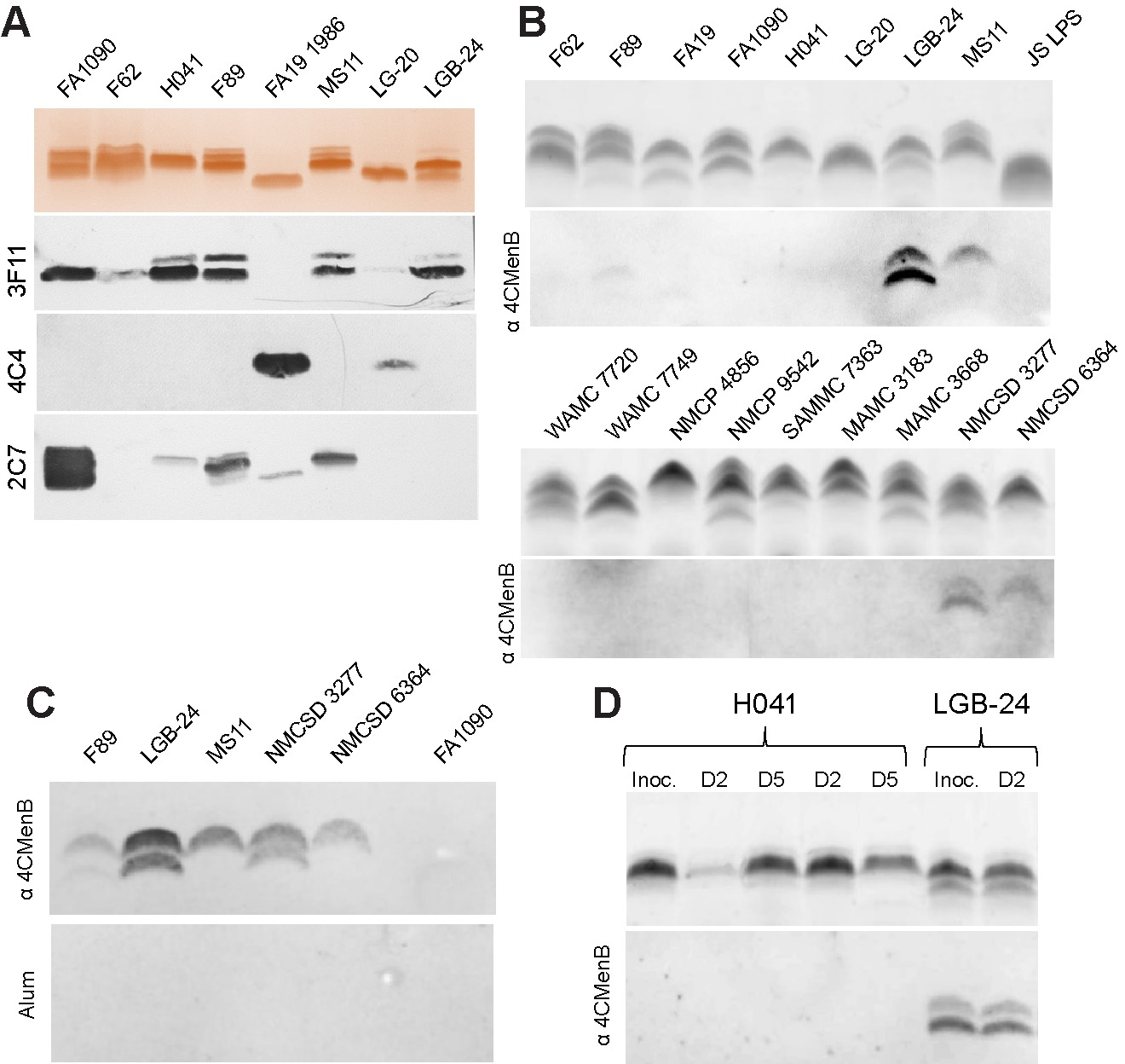

Supplement: S4 Fig — Proteinase K-treated bacterial extracts from 4 laboratory strains and 13 clinical isolates were resolved on 16% Tricine gels and stained with silver stain (top blot, panel A) or Emerald green (top blots, panels B and D), or electroblotted and probed with the following: (A) Mabs 3F11, 4C4 and 2C7, which recognize Ng LOS epitopes (bottom panels). Note that FA19 1986 is a variant of FA19 (panel B) that has a phase-off lgtA gene that results in truncation of the LOS to a single 3.6 kDa species [95]. (B) Pooled IP250 4CMenB antisera (bottom panels). The doublets in LGB-24 and NMCSD 3277 and single LOS species in MS11 and NMCSD 6364 that were recognized by the antiserum are distinct from the LOS species identified by the Mabs shown in Panel A. (C) Western blots against LOS from strains with 4CMenB-reactive bands incubated with pooled sera from 4CMenB-immunized mice versus mice given Alum only. (D) Emerald green-stained (upper panel) LOS from Ng strain H041 and LGB-24 (positive control) used to inoculate mice (Inoc) and from vaginal cultures collected on days 2 and 5 of infection. No change in the LOS species or 4CMenB reactivity was observed during infection by these strains. (TIF) [file ppat.1008602.s005.tif]

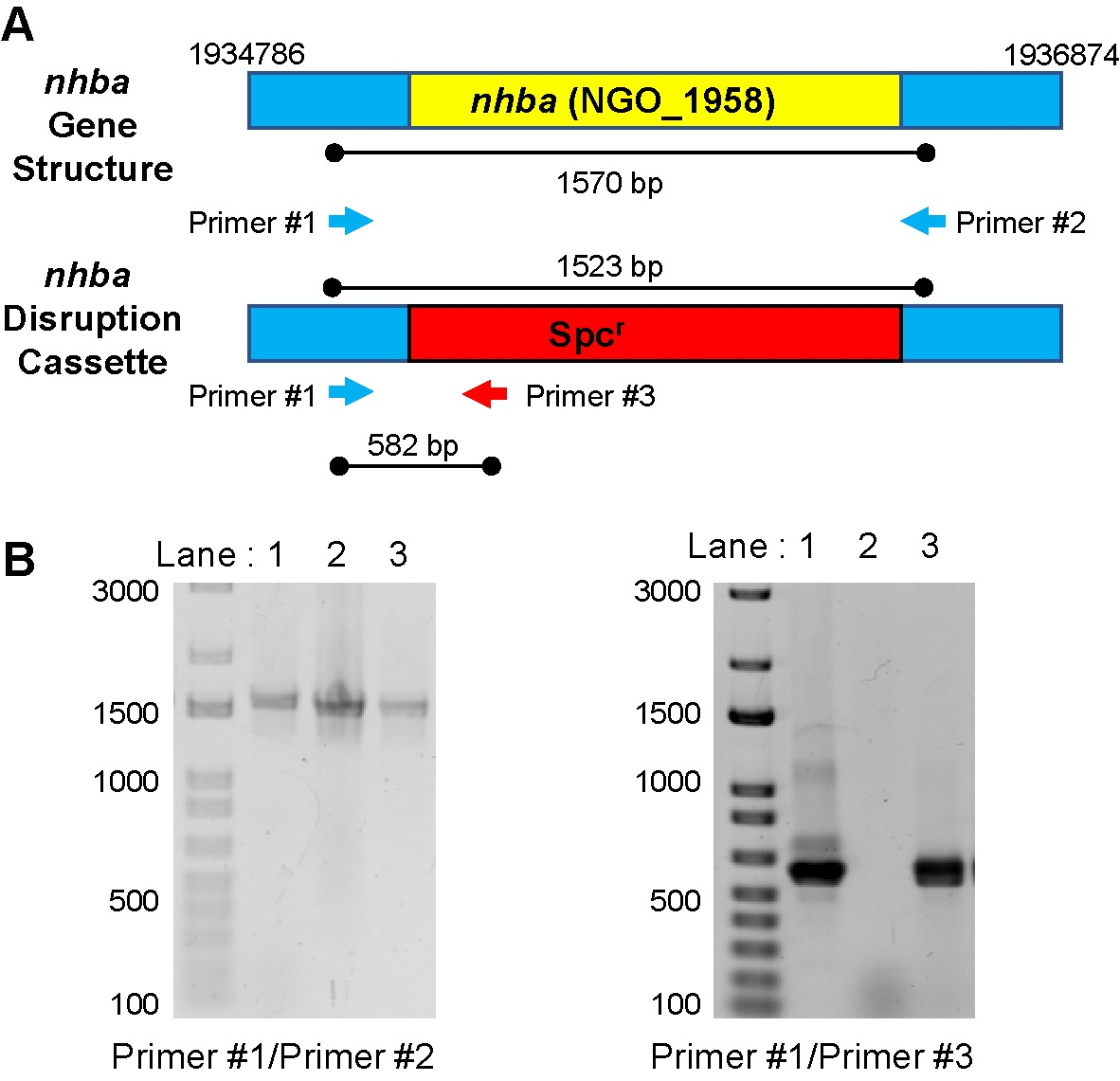

Supplement: S5 Fig — (A) Schematic of the synthetic Ng nhba disruption cassette showing 400 bp homology arms 5’ and 3’ from the nhba Ngo_1958) coding sequence, which is completely removed and replaced with a Spcr gene. The approximate location of primers used for polymerase chain reaction to detect the disrupted gene and he predicted size of the amplification products is noted. (B) Disruption cassette (Lane 1), and genomic DNA from FA1090 (Lane 2) and FA1090-Δnhba (Lane 3) were used as template for PCR with the indicated primers. The products of the amplification reactions were run on 1X TBE agarose gels and visualized with ethidium bromide staining. Oligonucleotide sequence primers were: Primer 1: ACGTTTTGTTTACCGCTGCC; Primer 2: TTCGGGGGCTTGTTTGATGA; Primer 3: CGTTGTCCCGCATTTGTAC. (TIF) [file ppat.1008602.s006.tif]
