# Supplementary material for: The serogroup B meningococcal outer membrane vesicle-based vaccine 4CMenB induces cross-species protection against Neisseria gonorrhoeae
Source: PLoS Pathog. 2020 Dec 8;16(12):e1008602. doi: 10.1371/journal.ppat.1008602 (PMC7748408; doi:10.1371/journal.ppat.1008602)
Supplement: Attachment — Submitted filename: Response to the Reviewers - Leduc et al., Oct 6, 2020.docx [file ppat.1008602.s007.docx]

Response to the Reviewers

**Part II – Major Issues: Key Experiments Required for Acceptance**

**Reviewer #1:** (No Response)

**Reviewer #2:** *A. The mice in the current study showed significant bacteridical activity against both serum sensitive and serum resistant gonococcal strains (Figure 3). In the Semchenko study, humans immunized with Bexsero did not develop a bactericidal response to the gonococcus. While it can be difficult to compare, the intensity of the immune response both in the number of antigens and types recognized in the murine response was considerably greater both by western blot and ELISA measurements than the human Bexsero response. The gonococcal literature since the mid-1970s has been replete with studies that have shown murine immune responses to pilus, porin and LOS epitopes which the human immune system either cannot or does not recognize. It would appear comparing the results of the Semchenko paper to this manuscript that we may be seeing the same situation in this model.*

We agree that a species-specific difference in the elicitation of bactericidal activity is important in that it confounds the ability to extend the prediction that bactericidal activity as a correlation of protection in human for this vaccine and perhaps other vaccines. We felt it was important to test human sera against the serum-sensitive strain F62 that we used in as the challenge strain because the strains used by Semchenko *et al.* (2019) and two reports by Beernink *et al.* (2019) were different, with the latter being a serum-resistant *Ng* strain. We found that unlike mice, 4CMenB-immunized humans don’t have significant bactericidal activity against the challenge strain based on the lack of a significant difference between the bactericidal_50_ titers of sera from 4CMenB-immunized and unimmunized humans. The results of these assays are shown in the **new Figure 10.**

We now state that with respect to bactericidal activity, the mouse model may not be predictive of vaccine efficacy in humans in lines 351-353: “We conclude from these results that the contribution of bactericidal activity in 4CMenB-induced protection against *Ng* infection may differ between mice and humans”.

Limitations to our study, however, that may have compromised our interpretation of both our human and mouse bactericidal data are i.) The lack of pre-immune sera with which the post-vaccination titers can be compared; expressing the data as a fold-change relative to pre-immune serum for each individual is a more sensitive way to detect differences among immunized and unimmunized individuals, and ii.) We did not determine the bactericidal activity of serum from individual mice (as questioned by Reviewer #1) due to inadequate sample volume. Looking at pooled sera is a less powerful way of identifying correlates of protection than comparing bactericidal activity and clearance rates in individual mice. Therefore the importance of the bactericidal activity we detected in pooled sera may or may not be as important as we suggested.

These limitations are now discussed the Discussion (lines 448-463): “In our studies here, bactericidal activity against the serum-sensitive challenge strain was detected in pooled serum from immunized mice. A more definitive analysis, however, would be to determine whether the bactericidal activity of serum from individual mice correlates with clearance rate, which we were unable to do due to insufficient sample volumes. We did not detect a significant difference in the bactericidal_50_ titers of serum from immunized humans compared to unimmunized individuals against the serum-sensitive challenge strain. If 4CMenB does indeed generate protective immune responses against *Ng* in humans, as is widely suspected based on ecological study data, these findings suggest bactericidal activity does not play a role in protection from *Ng* infection in humans. Comparison of post-vaccination titers to that of pre-immune serum from the same individual, which was not done in our study, is a more sensitive way to detect differences among immunized and unimmunized individual. A systematic assessment of the bactericidal activity of human immune serum against a diverse panel of *Ng* strains may also be warranted due to differences in susceptibility to complement and the expression level of surface components that could cause strain-specific variation in the susceptibility to 4CMenB immune sera from humans. Further investigation of the immune responses induced by 4CMenB in mice and vaccinated humans will continue to define mechanisms of vaccine-mediated protection in both species”.

Upon further exploring the issue of species-specific differences in bactericidal responses, we also state on lines 411-414 “More recently, infant rhesus macaques have been employed to study immune responses to *Nm* OMVs, and are more predictive than mice in assessing whether these vaccines induce bactericidal antibodies against *Ng* perhaps due to species-specific differences in TLR-4 recognition of lipid A within these vaccines [40]”.

**Reviewer 2, II. Part A,** continued: *“The gonococcal literature since the mid-1970s has been replete with studies that have shown murine immune responses to pilus, porin and LOS epitopes which the human immune system either cannot or does not recognize. It would appear comparing the results of the Semchenko paper to this manuscript that we may be seeing the same situation in this model”.*

We aren’t sure if the reviewer is referring to studies on immunized individuals or naturally or experimentally infected individuals. Since the time of those studies, *Ng* has been shown to employ several immunosuppressive pathways that affect the adaptive humoral response and therefore, the lack of, or reduced antibody response against *Ng* surface molecules in the context of infection in humans may not be unexpected. Immunization of humans or mice, particularly with purified proteins or OMV from related species, on the other hand, may result in a robust antigen-specific antibodies that are not induced during infection. We briefly raise this possibility in the Discussion, Lines 488-491:“The gonococcus is well-adapted to avoiding protective responses, and while serum from 4CMenB-immunized human serum did not recognize MtrE by western blot, an MtrE subunit vaccine may induce a specific protective antibody response in humans by not being presented in the context of an OMV”.

**Reviewer 2, II, Part B*.*** *For the results obtained in this model to be accepted as valid surrogates of the human response, it would be necessary to take the results from the murine system and demonstrated they have meaning in a human system. One possible way to do this would be to demonstrate that an anti-mtrE response is a significant part of the human response to Bexsero. Semchenko demonstrated a robust Neisseria Heparin binding antigen response in Bexsero immunized human sera by ELISA. The authors should consider taking at least one of the three antigens which appear to be potential vaccine candidates in the mouse model and show that antibodies to it, are present in Bexsero immunized human sera. Wang and Associates (J. Infection, 2018, 71:191-224) showed that mice immunized with mtrE surface exposed peptides showed protection in the same murine model of gonococcal infection. Using these peptides, the authors could address this issue directly if they want to prove that their model useful in identifying potential human vaccine candidates.*

We did not do ELISAs as suggested with purified MtrE or MtrE peptides due to lack of reagents and the time-limit of resubmission; instead we state more clearly that antibodies to MtrE were readily detected in 4CMenB-immunized mice, but were not in immunized human sera by western blot. We did conduct new experiments, however, using human serum samples to show that *Ng* PilQ is recognized by serum from both 4CmenB-immunized humans and mice, and that NHBA is recognized by immunized mice by doing western blots with murine immunized serum against wild-type FA1090 and an isogenic *nhba* mutant of this strain. These data are shown in the **new Figures 7, 8 and 9, Fig S5.**

These new data and the heterogeneity of immune responses in mice and humans are also now discussed in the abstract (lines 56-59): “Antibodies from vaccinated mice recognized several *Ng* surface proteins, including PilQ, BamA, MtrE, NHBA (known to be recognized by humans), PorB, and Opa. Immune sera from both mice and humans recognized *Ng* PilQ and several proteins of similar apparent molecular weight, but only mouse serum recognized MtrE. Pooled sera from 4CMenB-immunized mice showed a 4-fold increase in serum bactericidal_50_ titers against the challenge strain; in contrast, no significant difference in bactericidal activity was detected when sera from 4CMenB-immunized and unimmunized subjects were compared” to alert the reader to the contribution of this paper towards defining similarities and differences in the mouse and human antibody response to this vaccine. We appreciate the critique that without a comparison to humans, the previous version of this work was lacking relevance to humans.

**Reviewer #3:** *As stated in the summary above, I feel that all of the major points and conclusions of this paper are well supported by the experimental evidence. I did not identify any necessary additional experiments.*

We appreciate this reviewer’s enthusiasm for this work.

**Part III – Minor Issues: Editorial and Data Presentation Modifications**

**Reviewer #1:**

*1. The graphs in Fig 3 should show the killing over the entire range of concentrations – 1:30 to 1:960 (note the graph shows a range of 120 to 1920, while methods mention dilutions from 1:30 to 1:960, so need to unify). Please also show each value at least for the samples that got NHS – the methods state 2-3 independent experiments. Were the differences statistically significant? Also, what do the data points represent – mean, median?*

The range stated in the methods was in error; the range of concentrations tested are reflected in the figure. We have added a panel to show the % survival for each of three independent experiments when incubated with immunized or unimmunized mouse serum at 1:480 (F62) or 1:240 (FA1090), and show that the differences tested between pooled immunized and unimmunized mice in the bactericidal titers against the two *Ng* strains are significant. **New Fig 3B.**

*2. The reason for pooling sera for bactericidal assays should be stated. This limitation will overlook heterogeneity in bactericidal responses across animals and should be discussed.*

We weren’t able to do bactericidal assays on individual mice due to limited sample volumes and agree that it is preferable. These data would also allow us to look for correlations between bactericidal activity and clearance rates. In conjunction with collaborators we are in the process of miniaturizing bactericidal titer assays and hope that we can produce an assay that would make the experiments described feasible. We agree that single animal resolution of titer combined with infection clearance rate data in each animal would provide the most robust test for correlation between bactericidal activity and protection from infection in this model.

We elaborate on this issue now in Lines 449-45: “A more definitive analysis, however, would be to determine whether the bactericidal activity of serum from individual mice correlates with clearance rate, which we were unable to do due to insufficient sample volumes”.

*3. The authors have made a considerable effort to look for antibody responses to LOS (Fig 5). The OMVs in Bexsero are detergent-extracted, therefore contain only trace amounts of LOS. The anti-LOS cidal Ab described in Ref 32 were elicited after immunization with LPS-containing vaccine (NOMVs or detoxified LPS conjugated to proteins). The anti-LPS cidal Ab seen in children who got the Chilean vaccine (detergent-extracted OMV, contains <1% LPS) were seen 6 or more months after the last dose of vaccine, suggesting that they developed as a result of subsequent meningococcal colonization. Thus, rationale provided for looking at LOS response (lines 217-8) is unclear. It is not surprising that there is a limited response to LOS (from the presented data its not discernable whether pre-immune or adjuvant sera also would have reacted with Ng LOS). Note that humans immunized with Bexsero barely mount an Ab response to meningococcal or Ng LOS over baseline (see Supp Fig 6 in Semchenko et al, Clin Infect Dis, Vol 69, Issue 7, p 1101–1111). I suggest this entire section on LOS be removed or stated as a single sentence. The corresponding sentence in the abstract (lines 46-7) should also be deleted and Table 2 modified accordingly.*

We appreciate this reviewer’s knowledge on *Nm* OMV vaccines, which has helped us avoid giving a poor rationale for this line of investigation. We have deleted the rationale from this paragraph and shortened the text. We tested serum from Alum-treated mice to show that the reactivity we see against the minor number of strains is not due to pre-existing antibody as suggested by the reviewer, and we moved the figure to the supplemental material (**New Figure S4**). We elect to keep the data in the manuscript because it might be useful to someone studying neisserial LOS structures.

*4. The discussion focuses excessively on justification of use of this mouse model, and the possible advantages of transgenic mice. These portions (lines 332-372) can be considerably shortened and some of the comprehensive, outstanding reviews by the authors cited instead. The discussion should instead focus on how these results relate to other similar work (please see below).*

We have shortened this section by not discussing host-restricted colonization receptors or metal-binding proteins in detail, but elected to not delete it because of the opposition we have encountered regarding using non-human systems to study *N. gonorrhoeae*. Because of this, it is important to us that we state the limitations of the model whenever we present so that the results can be interpreted with this in mind.

*5. There should be a discussion of how these results compare to results reported by Semchenko et al, who examined Ng cross-reactive antibodies in humans given Bexsero. Were similar Ng proteins identified by immune mouse and human sera? Agreeably, the cross-reactive proteins, with the exception of NHBA, were not fully characterized in that study but it would be worth commenting on differences in molecular weights of the target proteins. It appears that the major Ng protein recognized in Bexsero immunized humans is in the ~25 kD range and against NHBA but does not appear to be the case here. These mouse/human differences are important and merit discussion.*

We have added new western blot data using serum from humans immunized with 4CMenB and compare the banding patterns with that of mice **(New Figs. 7, 8 and 9).** We have used arrows to point out bands that seem to be shared and those that are “uniquely” recognized by mouse antisera. The reviewer is correct that a very prominent band is present in the western blot with immunized human serum in the Semchenko paper. Upon inspecting our new western blots, some of which were run to include this region of the gel, unlike some of the earlier ones, a band of ~21 kD appears in westerns probed with immunized mouse serum (**Fig. 7A**). This band was also detected by IgG and IgA in vaginal washes from immunized mice (**Fig. 4B**), and with immunized human serum **Fig. 8A**.

We have added the detection of this band in the Results and elaborate about this in the Fig. 7 legend. Lines 1149-1152: “The ~ 20 kD band that is recognized by both human and mouse serum is similar in apparent molecular weight to a band that was strongly recognized by serum from a 4CMenB-immunized individual in Semchenko *et al.* [30]. A band of this molecular weight was also intensely recognized by IgG and IgA in vaginal washes from 4CMenB-immunized mice (Fig. 4B)”.

We note here (but not in the manuscript) that we see more discrete bands detected by the human immune serum, and possibly more bands, than that which are visible in the figures in the Semchenko paper. This is curious to us as well, but technical differences are the likely reason. Semchenko et al. used serum at a 1:4,000 dilution; our westerns are with a 1:500 dilution, and the substrate detection methods were different.

*6. Minimal bactericidal activity versus FA1090 was observed with serum from macaques immunized with Bexsero when tested at a 1:5 dilution (Beernink et al, mBio. 2019 Jun 18;10(3):e01231-19.). Humans given MenB-4C also do not show bactericidal activity against FA1090 (Beernink et al. J Infect Dis. 2019 Apr 1; 219(7): 1130–1137). Possible reasons for differences in the serum bactericidal responses across the species should be discussed*.

We have added this information to the discussion and are grateful and a little abashed to be pointed in the direction of this very nice literature. The species specificity with regard to bactericidal responses is important and this information will be useful to us in the future.

See lines 341-349: “Bactericidal antibodies against one or more of these proteins may accelerate clearance of *Ng* in the mouse model as suggested by the presence of bactericidal activity in serum from immunized mice (Fig. 3). Others have detected no or very low bactericidal activity in individuals vaccinated with 4CMenB- or NOMV-FHbp, which is an improved OMV-based serogroup B vaccine [30, 40, 41], while serum bactericidal activity against *Ng*, in contrast, was readily detected in immunized mice [40]. The strain used in two of these studies [40, 41] was a highly serum-resistant strain, and therefore here we assessed the bactericidal activity of serum from 4CMenB-immunized individuals against the serum-sensitive challenge strain used in our *in vivo* protection experiments”.

And lines 407-414: “Meningococcal vaccine development has been aided by the use of mice and rabbits to test whether candidate vaccines cause adverse effects or induce bactericidal antibodies against *Nm* [51, 52], and improved mouse and infant rat bacteremia models have been used to measure the efficacy of candidate *Nm* vaccines in eliminating *Nm* from the bloodstream [48, 53, 54]. More recently, infant rhesus macaques have been employed to study immune responses to *Nm* OMVs, and are more predictive than mice in assessing whether these vaccines induce bactericidal antibodies against *Ng* perhaps due to species-specific differences in TLR-4 recognition of lipid A within these vaccines [40].”

*7. Lines 386-389 “…; however, eleven other promising… (unpublished data)”. This part of the sentence should either be substantiated with data or deleted. This reviewer acknowledges that vaccines may function through mechanisms other than complement-dependent killing. Reports of high cidal titers need to be interpreted cautiously. This is because mice and rabbits often develop natural bactericidal Abs vs Ng with age and bactericidal assays reported for gonococcal vaccine candidates sometimes lack critical age-matched adjuvant controls, and thus does not account for the effect of these natural cidal Ab (eg, PMID: 27141096; PMID: 27895130). This study demonstrates baseline bactericidal activity in the pooled serum from unimmunized age-matched mice – a key control.*

We have deleted the unpublished data about other candidate vaccines and we thank the reviewer for their insight regarding the need for age-matched controls, which will help us in future studies.

*8. Line 71 – 87 million new infections (Rowley et al, Bull World Health Organ 2019;97:548–562P) –* New number of infections and reference added

*9. Line 127 – NHBA (not NHBP)* - corrected

*10. Was the NHS from Pel-Freez IgG and IgM depleted?* Yes, we have added this information to the Methods.
 *11. What dilution of serum was used in S1 Fig, panel C?* 1:5,000, added.

*12. Please also show the Area Under Curves for each individual mouse experiment (S2 Fig).* New panels added (Fig. S2C and F).

*13. Minor details/discrepancies in cidal methods in methods vs Fig legends – methods state 100-400 CFU used, legend states 10^4 bacteria. Time of incubation – 55 min in methods, 45 min in Fig legend.* The conditions were 10^4^ CFU and 55 min. This has been corrected so that it is uniform in both places.

*14. Line 316 –The meningococcal group C outbreak described in Ref 44 occurred at Fort Dix, NJ (not Fort Ord, CA).* We thank the reviewer for catching this error and have revised the sentence and included an appropriate reference.

*15. Lines 328-329 – only the 3 doses were similar, not the regimen. The vaccine preparation and the dosing interval were both different. Suggest deleting this sentence*. Agreed; we have deleted this sentence.

Reviewer #2: *(No Response)*

Reviewer #3: *I have no additional editorial suggestions.*

*-------------*

Changes made in response to Journal rules

*1. We noticed that you used “data not shown” in the manuscript, we do not allow these references. The PLOS data access policy requires that all data be either published with the manuscript or made available in a publicly accessible database. Please amend the supplementary material to include the referenced data or remove the references. Please make a note of this change in your response to reviewers.*

We removed the sentence (line 224): Serum from PBS- or Alum-treated control mice did not recognize any *Ng* proteins (data not shown). We feel that to show those data in this manuscript to which we have added 4 new figures and a new supplemental figure is not necessary, particularly since the Alum only and PBS-treated control mice are included in the ELISA data.

Discussion of MtrE conservation (lines 481-483) – We left the statement (“Importantly, the residues encompassing the two short surface-exposed loops of the MtrE monomer (residues 92-99 and 299-311 [65]), which are highly conserved among *Ng* strains, are identical to those from MtrE expressed by *Nm* strain MC58”) but removed the “(data not shown)” because the reader can look up reference 65 and do the comparison themselves if they wish to do so.

*2. We do not publish any copyright or trademark symbols that usually accompany proprietary names, eg (R), (C), or TM  (e.g. next to drug or reagent names). Therefore please remove all instances of trademark/copyright symbols throughout the text, including Bexsero® on page 2,3,6,8,15 and Trumenba® on page 15,18.*

Done

*3. Thank you for including your ethics statement: Serum specimens from the Department of Defense Serum Repository: The Armed Forces Health Surveillance Branch, Defense Health Agency, Silver Spring, Maryland [serum specimens 2016-2019; release dated 8/14/2019].. Please include: A statement that formal consent was obtained (must state whether verbal/written) OR the reason consent was not obtained (e.g. anonymity)*

We have revised this section to include a statement regarding formal consent (See bold font below): “Serum from healthy human subjects was collected as part of ongoing Department of Defense public health serosurveillance (PMID: 12453804). Serum was requested from deidentified subjects who had been previously immunized with the recommended 2 doses of 4CMenB were collected as part of an ongoing U.S.  Defense Health Agency (DHA), Immunizations Healthcare Division (IHD) *in vitro* investigation of the human immune response against Ng after 4CMenB immunization (IHBISP_18_017). Serum specimens were collected 2-16 months after last 4CMenB immunization for the immunized samples. Serum from non-immunized controls were also available from the IHB funded study. **Human serum was collected as part of routine public health surveillance and therefore consent for this study was not available. However, samples provided for this work were deidentified (anonymous).** Use of the human serum was approved by the USUHS Human Subjects Research Program Office.    Serum specimens from the Department of Defense Serum Repository: The Armed Forces Health Surveillance Branch, Defense Health Agency, Silver Spring, Maryland [serum specimens 2016-2019; release dated 8/14/2019]”.
